# B cell M-CLL clones retain selection against replacement mutations in their immunoglobulin gene framework regions

**DOI:** 10.3389/fonc.2023.1115361

**Published:** 2023-03-16

**Authors:** Hadas Neuman, Jessica Arrouasse, Ohad Benjamini, Ramit Mehr, Meirav Kedmi

**Affiliations:** ^1^ The Mina and Everard Goodman Faculty of Life Sciences, Bar Ilan University, Ramat Gan, Israel; ^2^ Division of Hematology and Bone Marrow Transplantation, Chaim Sheba Medical Center, Ramat-Gan, Israel; ^3^ Sackler School of Medicine, Tel-Aviv University, Tel-Aviv, Israel

**Keywords:** antibody, B lymphocytes, chronic lymphocytic leukemia (CLL), high-throughput sequencing (HTS), immunoglobulin (Ig), lineage trees, somatic hypermutation (SHM), machine learning (ML)

## Abstract

**Introduction:**

Chronic lymphocytic leukemia (CLL) is the most common adult leukemia, accounting for 30–40% of all adult leukemias. The dynamics of B-lymphocyte CLL clones with mutated immunoglobulin heavy chain variable region (IgHV) genes in their tumor (M-CLL) can be studied using mutational lineage trees.

**Methods:**

Here, we used lineage tree-based analyses of somatic hypermutation (SHM) and selection in M-CLL clones, comparing the dominant (presumably malignant) clones of 15 CLL patients to their non-dominant (presumably normal) B cell clones, and to those of healthy control repertoires. This type of analysis, which was never previously published in CLL, yielded the following novel insights.

**Results:**

CLL dominant clones undergo – or retain – more replacement mutations that alter amino acid properties such as charge or hydropathy. Although, as expected, CLL dominant clones undergo weaker selection for replacement mutations in the complementarity determining regions (CDRs) and against replacement mutations in the framework regions (FWRs) than non-dominant clones in the same patients or normal B cell clones in healthy controls, they surprisingly retain some of the latter selection in their FWRs. Finally, using machine learning, we show that even the non-dominant clones in CLL patients differ from healthy control clones in various features, most notably their expression of higher fractions of transition mutations.

**Discussion:**

Overall, CLL seems to be characterized by significant loosening – but not a complete loss – of the selection forces operating on B cell clones, and possibly also by changes in SHM mechanisms.

## 1 Introduction

Chronic lymphocytic leukemia (CLL) is the most common adult leukemia and stands for 30–40% of all adult leukemia cases (1, 2), and 7% of newly diagnosed cases of non-Hodgkin’s lymphoma (3). B-CLL (henceforth referred to as simply CLL) is a chronic B-cell malignancy, which typically affects elderly people, progresses gradually over many years, and involves substantial innate and adaptive immune system perturbations. Adaptive response impairments include down-regulation of T-cell function and defects in antibody-dependent cellular cytotoxicity, and in B cells – hypogammaglobulinemia and alterations in cell-cell contact and cytokine release, all of which may contribute to the overall immune suppression observed in patients (4). Indeed, during the COVID-19 pandemic, fatality rates for CLL patients were 16.5-fold more than the median population fatality rates reported worldwide, and even higher in older patients (5).

It has long been known that CLL genomes show heterogeneity between patients (6, 7), and that CLL clinical manifestations range from very indolent to aggressive disease (1). One partitioning of CLL is based on “stereotypic BCRs”, identified by the IgHV gene CDR3 region amino acid sequence; stereotypic BCRs can be assigned to 30% of CLL cases, and were associated with prognosis (8, 9). More importantly, CLL tumors are classified into two subgroups based on the presence of somatic hypermutations in their IgHV, where CLL patients with little to no SHM (98% IgHV sequence homology to germline) are defined as unmutated CLL (U-CLL), and CLL with SHM (less than 98% IgHV sequence homology) are defined as mutated CLL (M-CLL) (10). M-CLL patients have a better prognosis than those with U-CLL, as U-CLL is considerably more aggressive and less susceptible to chemo-immunotherapy (2, 8). This manuscript focuses solely on M-CLL (henceforth referred to simply as CLL). Although the mutational imprint on CLL cell IgHV genes has first been considered static, there is now clear evidence that, in a subgroup of cases, rearranged Ig genes are subject to ongoing mutational pressure (8). In such cases, the study of CLL clonal dynamics using Ig gene high-throughput sequencing (HTS) can yield important insights.

Since 2008, Adaptive Immune Receptor Repertoire HTS (AIRR-seq) has generated data sets of up to billions of reads (11, 12), and has, indeed, led to new insights into affinity maturation. BCR-seq has many applications (13), including broadly neutralizing antibody identification (14), vaccine response studies (15), B-cell migration and development tracking within the body (16) and disease diagnosis (17). In particular, Stamatopoulos and colleagues used HTS to sequence more than 200 CLL patient repertoires and demonstrated that one quarter of the CLL patients include multiple clones with unrelated, productively rearranged IgHV genes (18). The extensive amount of data that arise from AIRR-seq can also be analyzed using machine learning (ML) methods, e.g. for classification of B cell subpopulations, “public” vs. “private” clones, and more (19–21).

A B cell clone is a cell lineage that includes all the descendants of a founder B cell, all of which share a unique IgHV rearrangement; clonal diversification is best modeled by lineage trees. IgHV gene SHM and selection – including those in malignant clones, if any – are more precisely analyzed on IgHV gene lineage trees, because mutations are more correctly defined relative to the closest known ancestors, and thus mutation counts – and all the analyses relying on them, including selection analysis, in which CDR3s can only be included if using lineage trees – and lineage tree topologies are more correct on lineage trees (22). Using lineage trees, Abraham and colleagues found evidence of intraclonal diversification of characteristic clones in light chain amyloidosis patients, concluding the pathogenic plasma cells are probably derived from a precursor population in which SHM is ongoing (23). Zuckerman et al. used lineage tree-based mutation analysis to find that follicular lymphoma (FL), diffuse large B cell lymphoma (DLBCL), and primary central nervous system lymphoma repertoires have similar mutation frequencies and do not undergo positive selection *for* replacement mutations in their CDRs (24), using the focused binomial test (25) rather than relying on previously published tests for selection, which have all been shown to generate false positives (26, 27). The transformation of FL into DLBCL has been followed using clonal lineage trees to show that, in some cases, therapy eradicates a DLBCL clone but a new one develops from remnants of the original FL clone (28, 29). Lineage tree analysis of dominant clones from mucosa-associated lymphoid tissue lymphoma showed higher diversification and longer mutational histories compared with chronic gastritis or with gastric DLBCL (30); gastric DLBCL may originate from gastritis, mucosa-associated lymphoid tissue lymphoma or *de novo*, and, like CLL, may sometimes contain more than one dominant clone (31). Green et al. used lineage trees to distinguish early versus late genetic events in follicula lymphoma (32). Béguelin and colleagues used lineage tree analysis to show evidence of reduced efficacy of affinity maturation in mice with EZH2 mutations, which initiate lymphomagenesis (33). Finally, Kedmi et al. showed that the use of lineage trees is necessary for detection of minimal residual disease in a DLBCL patient, prior to its detection by PET-CT (34). In this work, we aimed to study the SHM and selection (if any) mechanisms that operate on CLL clones using IgHV gene lineage tree-based analyses and machine learning methods, which to the best of our knowledge have never been previously applied to CLL. Such analysis can yield novel insights, as demonstrated by our most important finding, i.e. that while CLL dominant clones undergo weaker selection *for* replacement mutations in their CDRs, they *retain* some selection (albeit weaker than that in healthy controls and non-dominant clones) *against* replacement mutations in their FWRs.

## 2 Methods

### 2.1 Datasets

IgHV gene sequences from peripheral blood samples of 16 M-CLL patients were obtained for routine diagnosis of mutated vs. unmutated CLL cases. Only M-CLL samples were chosen for this study. Sample data are summarized in **Table 1**. Buffy coats were taken, and DNA was extracted directly from the buffy coat of each sample. IgHV gene libraries were produced using the LymphoTrack^®^ kit (Dx IGHFR1 Assay Panel for MiSeq, Catalog #91210039, *In vivo*scribe, San Diego, CA, USA). Sequencing was performed using the MiSeq V300 kit (Illumina, San Diego, CA, USA) according to the manufacturer’s protocol. The use of the resulting IgHV sequence data (without any clinical or other identifying data) was approved by the Sheba Medical Center and Israeli Ministry of Health review boards.

**Table 1:** CLL patient dataset.

Sample name	# Sequences in raw data	# Unique sequences after processing	# Clones	# Clones with 2 or more sequences
**INDEX2_S1**	1,040,682	9,373	182	100
**INDEX3_S2**	489,773	8,456	365	343
**INDEX4_S3**	763,818	4,014	53	17
**INDEX5_S4**	546,554	4,654	40	24
**INDEX6_S5**	1,147,666	15,969	684	652
INDEX7_S6*	657,167	25,750	2,473	1,840
**INDEX8_S7**	1,421,821	11,620	52	38
**INDEX9_S8**	382,498	2,330	21	12
**INDEX10_S9**	1,167,134	56,998	6,797	6,390
**INDEX12_S10**	736,105	9,554	96	87
**INDEX13_S11**	967,315	6,056	86	70
**INDEX14_S12**	686,510	8,563	26	16
**INDEX15_S13**	726,865	6,306	70	58
**INDEX16_S14**	547,240	2,688	243	129
**INDEX18_S15**	749,263	3,817	20	15
**INDEX19_S16**	945,151	4,896	70	55
**Overall**	12,975,562	181,044	11,278	9,846

* This sample contained several large clones, so that the dominant clone could not be identified with certainty; hence this sample was omitted from the study.

For comparison with CLL patient repertoires, we used IgHV sequences from three blood samples of healthy individuals (35), which are publicly available, and were downloaded by us as part of a different study. Since CLL is most common in elderly patients, we chose the samples of the three eldest healthy individuals for this comparison; healthy control (HC) sample data are summarized in **Table 2**. For negative controls in the selection analysis (see below), we used lineage trees composed only of sequences containing a frame shift, taken from the same CLL patients, as these sequences most likely represent non-productively rearranged, non-expressed alleles.

**Table 2:** Healthy repertoire samples.

Sample name	Sex	Age	# Unique sequences after processing	# Clones	# Clones with 2 or more sequences
**H45_3**	F	45	169,243	67,346	24,841
**H45_4**	F	45	257,571	171,620	35,946
**H50_7**	F	50	118,472	50,453	18,404
**Overall**			545,286	289,419	79,191

### 2.2 Data processing steps

We preprocessed the sequences using pRESTO version 0.5.13 (36). The preprocessing included assembly of paired ends and quality filtering by (i) trimming low quality edges, (ii) filtering out reads with an average Phred score lower than 25, and (iii) masking bases with Phred scores lower than 20. Sequences with more than 10 masked or missing bases were removed. Since the sequencing kit manufacturer does not consent to reveal the primer sequences, we removed 30 nucleotides from both ends of each sequence. Next, identical sequences were collapsed, and only sequences with two copies or more were selected for analysis; this is standard practice meant to reduce the chance of including PCR and sequencing errors in cases such as this, where unique molecular identifiers UMIs were not used. Further precautions we took to minimize such errors were: (a) Using only one copy of each set of identical sequences in the lineage tree analysis; sequence copy numbers weren’t used in any of our analyses. (b) Omitting “clones” that contain only one unique sequence (regardless of its copy number) from the analysis.

**Figure 1:**
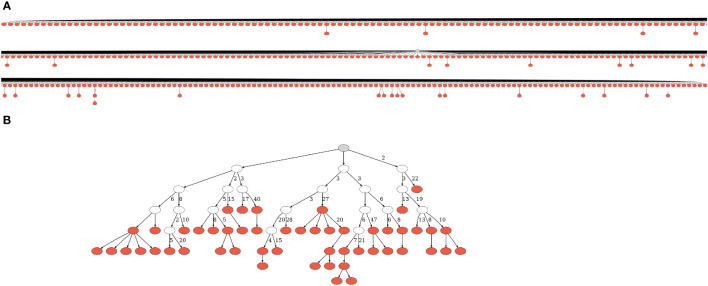
Lineage trees from CLL patients. **(A)** One of the smallest trees from expanded, dominant clones. Due to its size, we had to split the figure into partly overlapping segments. **(B)** One of the largest trees from presumably normal, non-dominant clones. A gray node represents the root, and a white node – a hypothetical split node. Numbers next to edges denote numbers of mutations; edges with no adjacent numbers represent one mutation. The trees were drawn using IgTreeZ (22) and Graphviz (40). More representative trees of all sizes are given in **Figure S1**.

We further processed the selected sequences using Change-O version 0.4.6 (37) and in-house custom scripts. The processing included annotation of the sequences with the IMGT/GENE-DB (38) reference germline sequences from July 1, 2021, and removal of sequences annotated as non-functional (those with frame-shifts or stop codons); dynamic clonal assignment according to V and J segment annotation and junction (CDR3) similarity (the numbers of clones in each sample are given in **Table 1**); and assessments of sampling depth and of the clonal size distributions of each repertoire. Putative germline sequences for each clone were created based on the same IMGT/GENE-DB database and the clonal consensus in junction regions, and clones with more than two unique sequences were sent to IgTree^©^ (39) for lineage tree construction. Sample Lineage trees are shown in **Figure 1** and **Figure S1**. Note that the only times a lineage tree node may represent more than one sequence is when these unique sequences differ by mutation(s) that fall in sequence margins, and these margins were further trimmed by IgTree^©^ because one or more sequences in the clone lacked information on those margins.

To focus on the malignant clones in CLL patient repertoires, we separated the largest (dominant) clone from each repertoire, assuming it is the malignant clone. As internal controls, we used the non-dominant clones from the same patients, under the assumption that these are normal B cell clones (although they may be reactive to the tumor itself). This assumption was based on the knowledge that all B cell populations are composed of clones; even naïve B cells divide a few times before settling into a resting state, and may later perform homeostatic cell divisions (41). One sample included several large clones, and hence was omitted from the study (**Table 1**), to avoid the possibility of including a second CLL clone in the “non-dominant” control group. The healthy control repertoires served as external controls; for the sake of studying SHM and antigen-driven selection, if any, clones that were reactive at the time of sampling are the most valuable controls.

### 2.3 Lineage tree-based analyses

#### 2.3.1 Tree-based mutation analyses

Lineage tree-based mutation analyses were performed using our program IgTreeZ (22), based on the linkage of tree nodes to their corresponding sequences. IgTreeZ traverses all tree nodes, counts all the observed mutations, and characterizes each mutation by its sequence location (CDR/FWR, based on IMGT region definitions (42)) and type (source nucleotide, transition/transversion, replacement/silent); if it was a replacement mutation, the program also characterized the pre- and post-mutation amino acids based on IMGT physicochemical amino acid classes (43, 44).

#### 2.3.2 Selection analysis

Selection analysis was done using ShazaM (37, 45), which is based on the focused binomial test (25). The numbers of silent and replacement mutations in the CDRs and FWRs for all sequences in each tree received from IgTreeZ were sent to ShazaM, together with the corresponding clonal germline sequence and the CDR3 length of each tree. Using ShazaM, we calculated the expected mutation frequency in each region of each sequence, estimated the selection strength for each tree, and compared the selection scores of the different lineage tree repertoires. CDR3s were included in the analysis by modifying ShazaM’s region definition parameter according to each tree’s CDR3 length and calculating the expected mutation frequency for each clonal germline sequence separately.

#### 2.3.3 Tree topology analysis

Seven graphical shape properties of IgV gene lineage trees were found to be most strongly influenced with B cell response parameters, such as activation, division, mutation and death rates and selection thresholds (46). The seven tree shape properties are: (i) trunk length (the number of mutations from the root node, which represents the pre-mutation sequence, to the first split node), (ii) the minimum root to leaf path (i.e. the minimum number of mutations per leaf), (iii) the minimum root to split node path (which equals the trunk when there is one), (iv) the number of children emerging from the root (a node’s “children” are defined here as those representing sequences that differ from the parent node by a single mutation), (v) the average number of children per node, (vi) the average distance from the first split node to any leaf, and (vii) the minimum fork to fork distance (that is, the distance between two consecutive splits on the same path). IgTreeZ (22) calculates these variables for each tree and enables us to and compared the results between groups.

#### 2.3.4 Tree drawing

To visually illustrate lineage tree shapes (**Figure 1** and **Figure S1**), we created drawings using the graph description language DOT, as implemented in Graphviz (40). Node (sequence) names were omitted for better tree visualization.

#### 2.3.5 Tree trunk removal

To exclude as much as possible of the pre-transformation mutation and selection history of each lymphoma clone from some of the analyses, we removed the trunks from the trees in all groups, and assigned the first split node of each “trunkless” tree to be the new root node. Trees that originally had no trunks were removed from the trunkless analyses, so the data are not biased, as such trees did not contain enough information regarding their diversification history. However, since the latter step left only three trees for analysis, we performed most analyses both with and without tree trunks and compared the results.

### 2.4 Statistical analyses

Comparisons between lymphoma lineage tree characteristics against those of healthy repertoires, which included more than 50,000 trees, were done based on the average measurements per patient/subject, to overcome the bias of the healthy control dataset being so much larger (in terms of numbers of trees) than the other datasets. For each comparison, the assumptions of normal data distribution and variance homogeneity were tested using the Shapiro test and the Levene test, correspondingly. If the data were normally distributed and had homogenous variances, Student’s t-test or its paired version were used. Otherwise, the non-parametric Mann–Whitney U-test, or the Wilcoxon test for paired comparisons, were used. To correct for multiple comparisons, we used Benjamini and Hochberg’s False detection rate (FDR) method (47). Only differences with p-values lower than the FDR-corrected α were considered to be significant.

### 2.5 Machine learning classification models

We used all the results of lineage tree-based mutation analyses of the CLL non-dominant and healthy control clones as input for our ML models. Data in all columns which included simple mutation counts were normalized by dividing them by the total number of mutations in each tree, to receive the *frequency* of each mutation type. Columns listing median and average replacement distances and CDR3 lengths were not normalized. We also excluded FWR1 mutation counts from the analysis, as it may be influenced by the sequencing. Since we had almost tenfold more healthy control clones than non-dominant clones in CLL patient samples (**Tables 1**, **2**), the dataset was balanced using the SMOTetomek algorithm (48) – a combination of oversampling the CLL data by synthesizing new examples based on the structure and composition of the real non-dominant clones using SMOTE (49), and under-sampling of the healthy control data using the TOMEK algorithm (50). Three ML models were built using Python’s Scikit-learn package (51) – a Support Vector Machine (SVM), a Random Forest and an XGBoost model. The F1-score, which is the harmonic mean of model precision and recall, was used as a model performance metric, in order to account for both measures.

## 3 Results

### 3.1 Dominant CLL clones undergo, or retain, more replacement mutations that alter amino acid physical properties

**Figure 2:**
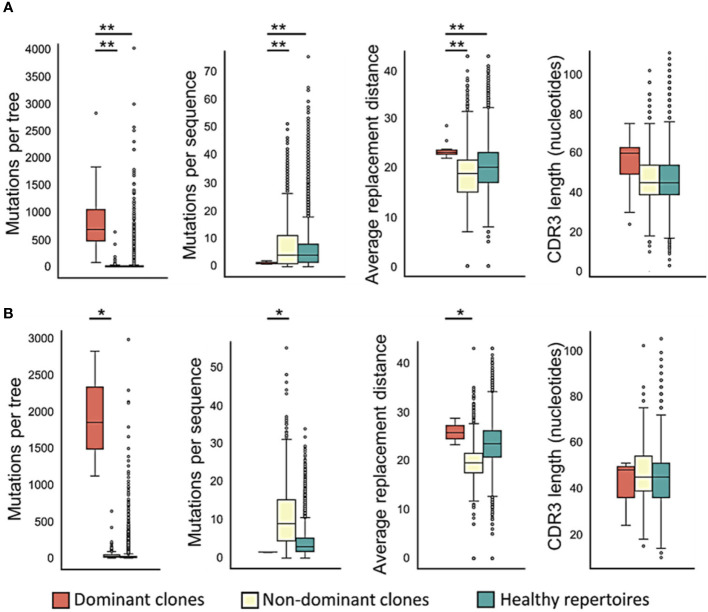
Dominant CLL clones undergo – or retain – more mutations, in particular replacement mutations, than non-dominant or healthy control clones. **(A)** Trees with trunks; **(B)** trunkless trees. The average physico-chemical distance was calculated between pre- and post- replacement mutation amino acids based on Sneath’s index (52). The paired T-test or the Wilcoxon paired test were used when comparing between dominant and non-dominant clones in the same patients, and Student’s T-test or Mann-Whitney test – between patient and healthy control clones, depending on whether the data were distributed normally or not. ∗p < 0.05, ∗∗p < 0.01.

To examine CLL clone diversification, we first compared trees of dominant and non-dominant clones in CLL samples (each group separately) with trees of healthy controls, and found that dominant CLL clones include significantly more mutations per clone than non-dominant clones in the same patients (**Figure 2A**, *p* < 0.01, Wilcoxon paired test and FDR correction), or than clones from healthy control repertoires (*p* < 0.01, Mann Whitney test and FDR correction). Since tumor clone lineage tree ‘trunks’ may contain mutations that had occurred prior to malignant transformation, we also performed all analyses on the trees after trunk removal, as described in the Methods section. The above-described differences were also found in the trunkless tree analysis (**Figure 2B**, *p* < 0.05 for both comparisons, Wilcoxon paired test and FDR correction); the higher p-values in trunkless analysis vs. analysis with trunks may result from the decreased numbers of data points due to the exclusion of original trunkless trees. In contrast, when we compared the numbers of mutations *per sequence*, we found that dominant clones have fewer mutations per sequence than non-dominant (*p* < 0.01, Wilcoxon paired test and FDR correction) and healthy repertoire clones (*p* < 0.01, Mann Whitney test and FDR correction). These differences were also found in the trunkless trees, with larger p-values. This results in the highly branched rather than “long” shape of the CLL trees (**Figure 1** and **Figure S1**), which, in our experience, is typical not only in CLL but also in other B cell GC-derived lymphomas. We hypothesize that the combination of high numbers of mutations *per tree* with low numbers of mutations *per sequence* result from having a population of malignant cells constantly dividing and generating new mutants, which do not get to mutate further because the cells still retain *some* selection against deleterious mutations, as further investigated and discussed below.

**Figure 3:**
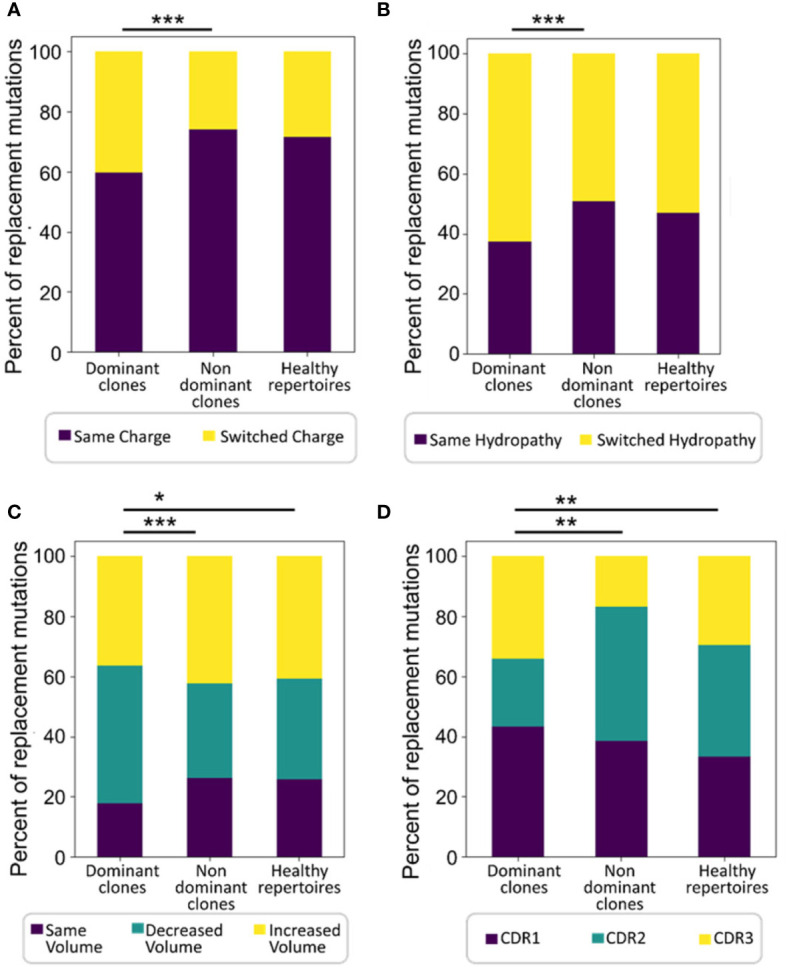
Dominant clones undergo or retain more replacement mutations that alter amino acid properties. Shown are percentages of replacement mutations in all trees that change the amino acid **(A)** charge, **(B)** hydropathy, or **(C)** volume, and **(D)** the distributions of mutations among CDRs in all trees. Significant differences were also found in mutations that change amino acid polarity, chemical group, and the tendency to donate and accept hydrogen (not shown). The Wilcoxon paired test was used when comparing between dominant and non-dominant clones in the same patients, and the Mann-Whitney test – between patient and normal healthy controls, as the data were not normally distributed. ∗p < 0.05, ∗∗p < 0.01, ∗∗∗p < 0.001.

The average physico-chemical distance between pre- and post-replacement mutation amino acid, measured by Sneath’s index (52), was larger in dominant clones than in non-dominant or healthy repertoire clones (both *p* < 0.01, Mann Whitney test and FDR correction). Indeed, comparisons of several individual components of the Sneath index – that is, the frequencies of changes in several different amino acid properties – revealed that dominant clones tend to undergo, or retain, more replacement mutations that alter the amino acid charge, volume, and/or hydropathy more often than non-dominant clones and healthy repertoires (**Figure 3**).

The excluded, original trunkless dominant trees tend to have significantly more mutations per tree (*p* < 0.01, paired *t* test), than the dominant clone trees with trunks (**Figure S2**). The numbers of mutations per tree were also higher in originally trunkless dominant trees compared to trunk-including trees (*p* < 0.01, Mann Whitney test). Finally, the average physico-chemical distance between pre- and post-mutation amino acids in replacement mutations was higher in originally trunkless dominant trees compared to trunk-including trees (*p* < 0.05, Mann Whitney test). The latter differences may be due to the time it took for each CLL clone to develop until the sample was taken. Since every mutation requires cell replication to be completed, slower-growing clones, whether normal, pre-malignant or tumor clones, will gather fewer mutations. In addition, as long as the cells are sensitive to some level of selection, cells with harmful BCR mutations will eventually die, and thus such cells will produce fewer progeny overall. Slower-growing tumors are also likely to be detected after growing for a longer time, as it would take longer for symptoms to manifest in the patient. As a result of all these considerations, we assume that earlier branches of slow-growing clones have a lower chance of being picked up in the sample, and thus slower-growing clones are more likely to have both longer lineage tree trunks. Overall, the results presented in this section demonstrate that M-CLL tumors have very heterogenous diversification histories, and the presence of trunks in most lineage trees of these clones suggests that they may have been subject to some degree of selection against harmful BCR mutations, not only before the malignant transformation but also following it, even up to the time of sampling.

### 3.2 Lineage tree topologies suggest that CLL dominant clones retain some sensitivity to selection

Next, lineage tree topologies were studied, as another way to examine clonal diversification; here, we only present lineage tree shape properties for which significant differences between groups were detected. Trees from dominant CLL clones were found to have significantly shorter trunks than trees from non-dominant clones in the same patients (**Figure S3A**; *p* < 0.01, Wilcoxon paired sample test and FDR correction) and from healthy controls clones (*p* < 0.01, Mann-Whitney *t* test and FDR correction). The minimum root to leaf path (i.e., the minimum number of mutations per leaf) and the minimum root to fork path were significantly shorter in trees from dominant clones than in those from non-dominant (*p* < 0.01 for both, Wilcoxon paired sample test and FDR correction) or healthy control clones (*p* < 0.05 and *p* < 0.01, respectively, Mann-Whitney *t* test and FDR correction). In the original simulation study described in the methods section on which our interpretations are based (46), these lineage tree “length” measures were inversely influenced by initial clone affinity and selection strength, which makes intuitive sense, because (a) the higher the initial affinity, the fewer mutations are needed (if at all) for the BCR to reach the optimal shape for binding its cognate antigenic epitope (i.e. where any mutation would decrease the affinity, see also (53)); and (b) the more stringent antigen-driven selection is, the fewer mutations will survive. It is harder to interpret the shapes of tumor clones; however, their shorter branches suggest that CLL cells retain *some* sensitivity to selection.

CLL clone lineage trees are not only shorter but also much more branched, as demonstrated by the following findings. The numbers of leaves (branch endpoint nodes) per tree were significantly larger in dominant clones, with a median of 537 leaves per tree, rather than 1 in non-dominant and healthy control clonal trees, as most normal B cell clones are represented in the peripheral blood by one or very few sequences. The numbers of children emerging from the tree root, and the average number of children per node, were significantly larger in trees from dominant clones than in those from non-dominant (**Figure S3B**; *p* < 0.01, Wilcoxon paired sample test and FDR correction) or healthy control clones (*p* < 0.05, Mann-Whitney *t* test and FDR correction). The median number of children emerging from the root was 342 descendant nodes in dominant trees and 1 descendant node in non-dominant and healthy control trees. In the original simulation study (46), these lineage tree “branching” measures were directly influenced by initial clone affinity – the higher the initial affinity, the more success in forming additional branches, as explained above – and the average number of children per node was inversely influenced by selection strength, again because selection would “trim” lower-affinity branches. To interpret the shapes of CLL clones, we should ignore initial (presumably pre-transformation) clonal affinity, and only refer to the highly branched shapes of the observed clonal trees. These shapes suggest that whatever selection acts on the IgHV mutants is weak enough to allow a constantly dividing and mutating tumor cell population to continuously replenish the dominant clone cells in the blood. Finally, the trunkless analysis showed similar trends to those in the trunk-including analysis (**Figures S3C, D**), though with lower statistical significance due to the smaller group sizes.

### 3.3 CLL dominant clones undergo weaker selection *for* replacement mutations in the CDRs, but retain selection *against* replacement mutations in the FWRs

**Figure 4:**
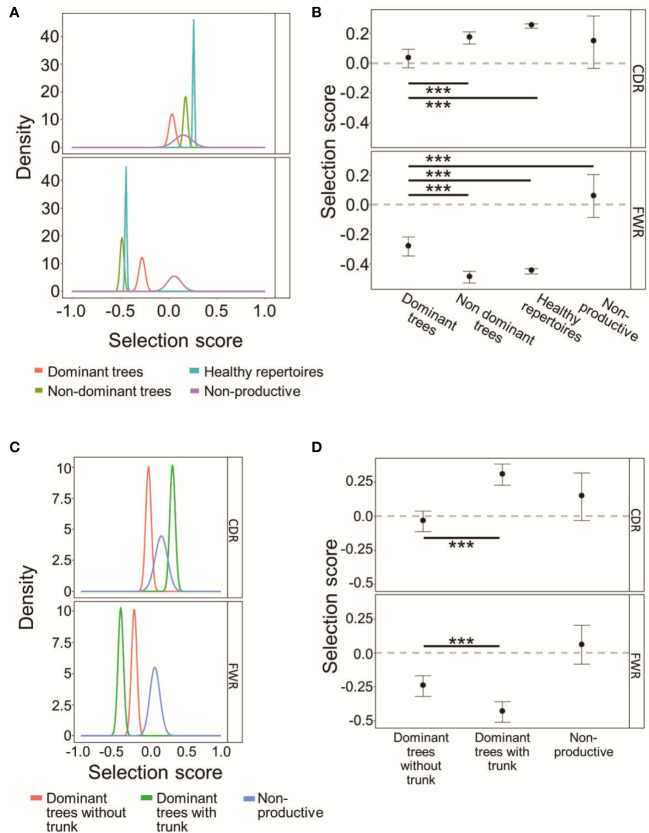
CLL dominant clones retain some selection *against* replacement mutations in the FWRs. **(A)** The probability density functions of the selection scores for dominant CLL clones in comparison to non-dominant clones in the same patients, or healthy donor clones, and to fully non-productive clones, calculated on the lineage tree-based mutation counts of the same data. Positive values indicate selection for, and negative values – selection against, replacement mutations. **(B)** Means and 95% confidence intervals of the selection scores plotted in **(A)**. **(C, D)** CLL dominant clones without trunks exhibit weaker selection than dominant clones with trunks, both for replacement mutations in the CDRs and against replacement mutations in the FWRs. **(C)** Same as **(A)** for *trunkless* trees. **(D)**. Means and 95% confidence intervals of selection scores of the selection scores plotted in **(C)**. The line at Selection Score=0 is shown to indicate when the results are indistinguishable from the case of no selection operating on the clones. Both graphs were plotted using ShazaM (37, 45) based on the focused binomial test (25). ∗∗∗p < 0.001, Student’s T-test with FDR correction for multiple comparisons.

To directly test which, if any, type of selection has been acting on the mutated CLL and control clones and to what extent, IgTreeZ mutation counts in the FWR and CDR regions were used as input for the ShazaM R package (37, 45). We also created a cohort of non-selected control clones by assigning all sequences in each repertoire – functional and non-functional – into clones, and constructing lineage trees from the clones that included only out-of-frame IgHV sequences, presumably representing un-productively rearranged, non-expressed IgHV alleles. Selection scores measured on all four clonal repertoires show that dominant clones undergo the weakest selection – or none at all – *for* replacement mutations in the CDRs, similar to the non-productive clones (**Figures 4A, B**), compared to that in non-dominant or healthy control clones (p < 0.001, Student’s T-test with FDR correction for multiple comparisons). In contrast, in the FWRs, dominant clones clearly undergo selection *against* replacement mutations (as their selection scores significantly differ from those of the non-selected clones; the latter have scores that do not significantly differ from the case of no selection, depicted by the zero line), although it is weaker than the same selection observed in non-dominant clones and in healthy repertoires (p < 0.001, Student’s T-test with FDR correction for multiple comparisons). Selection scores in non-dominant clones were similar in both CDRs and FWRs to those in healthy repertoires. Overall, these results suggest that the selection that operates on CLL clones is not completely abolished, but is certainly different from that in normal repertoires. The selection *against* replacement mutations in the FWRs may represent a need for (at least partial) maintenance of the structural integrity of the B cell receptor, as discussed below.

Dominant CLL clone trees with trunks removed still seem to undergo selection *for* replacement mutations in the CDRs, and selection *against* replacement mutations in the FWRs, similarly to trees from healthy repertoires (**Figures 4C, D**). Dominant trees that originally had no trunks, however, undergo weak – if any – selection in the CDRs, with indistinguishable scores from those measured on the fully non-productive clones. These results further illustrate CLL tumor heterogeneity, and emphasize the need for trunk removal from tumor clone trees, as many replacement mutations in tree trunks must have been selected (for or against), so including the pre-transformation mutation history in lymphoma clone analysis may confound the results.

### 3.4 Machine learning reveals potential SHM impairments even in *non*-dominant patient clones

In the past, we have shown that IgTreeZ extensive mutation counts can be used as input for ML models, to further elucidate the mutation mechanism in DLBCL clones (22). In the current study, we compared only patient *non*-dominant clones to healthy control clones; malignant clone data were not included in the ML models, as the purpose of the ML models was to identify traces of potential CLL patient-specific (rather than tumor-specific) impairments in SHM or antigen-driven selection, rather than to distinguish between patient and healthy control clones.

To perform the most unbiased analysis we could, we first normalized the mutation counts by dividing the specific mutation counts by the total number of mutations in each tree to receive the *frequency* of each mutation type. Second, since our dataset was extremely imbalanced, with almost tenfold healthy control clones than non-dominant clones in CLL patient samples (**Tables 1**, **2**), we balanced the dataset using the SMOTetomek algorithm (48). Third, we built three different machine learning models – a Support Vector Machine (SVM), a Random Forest and an XGBoost model – to classify the revised datasets.

**Figure 5:**
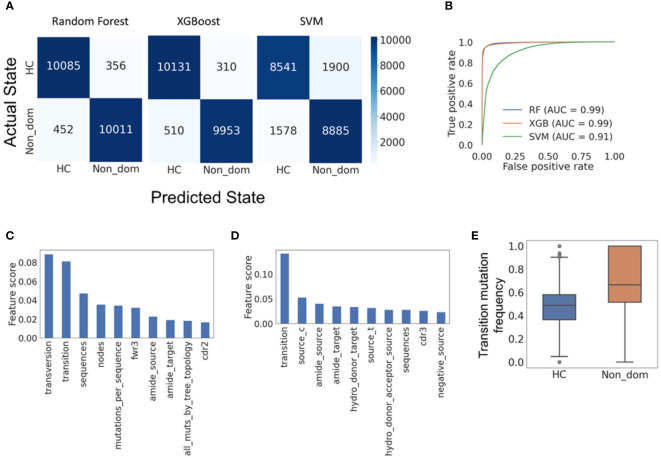
Machine learning models reveal features distinguishing CLL non-dominant from healthy control clones. **(A)** Confusion matrices for the Random forest, XGBoost and SVM models, respectively, all showing high accuracy in classification of the clones. **(B)** Receiver operating characteristic (ROC) curves for the three models. Such plots give the true-positive rate (a.k.a. sensitivity, recall or probability of detection) vs. the false-positive rate (a.k.a. the probability of false alarm, or 1 – the specificity). The larger the area under the curve (AUC), the better the model performance is. **(C)** Random forest feature importance scores (proportions of the influence of each feature out of the summed influences of all features), showing which model features are responsible for most of the separation between data groups. **(D)** XGBoost feature importance scores. **(E)** The frequencies of transition mutations in the two data groups; a p-value could not be determined, as the group size was larger than 5000. Confusion matrices and ROC curves were created using Python’s scikit-learn package. HC – Healthy controls, Non_dom – Non-dominant CLL clones. RF – Random Forest. XG – XGBoost, SVM – support vector machine. Transversion, Transition – transversion or transition mutation frequency, respectively. Fwr3 – the frequency of mutations in the FWR3 region out of all mutations. Amide_source (target) – the frequency of amide amino acid in pre-(post-) replacement mutation amino acids. Cdr2 (cdr3) – the frequency of mutations in the CDR2 (CDR3) region out of all mutations. Source_c(t) – the frequency of cytosine (thymine) among all pre-mutation nucleotides. Hydro_donor_target – the frequency of hydrogen donating amino acids among post-mutation amino acids in replacement mutations. Hydro_donor_acceptor_source – the frequency of hydrogen donating and accepting amino acids among pre-mutation amino acids in replacement mutations. Negative_source – the frequency of negative amino acids among pre-mutation amino acids in replacement mutations.

All three classification models exhibited very high accuracy; Random Forest presented the best performance with F1-scores of 0.962 and 0.961 for HC and non-dominant trees, respectively, and SVM the worst, with F1-scores of 0.831 and 0.836 for HC and non-dominant trees (**Figure 5A**). XGBoost performed almost as well as Random Forest (**Figure 5B**). To assess the relevance of specific input parameters to this classification – and thus to learn which features of SHM are specific to non-dominant clones from CLL patients rather than to their tumors – we calculated the feature importance scores of the Random Forest and XGBoost models. The transition mutation frequency was found to be the best predictor, accounting for 0.08 of the separation in Random Forest (**Figure 5C**) and 0.15 of the separation in XGBoost (**Figure 5D**). Indeed, transition mutation frequencies in the CLL non-dominant clones tended to be higher than those in healthy controls (**Figure 5E**). Overall, these results suggest either that the presence of CLL malignant clone(s) influences SHM or selection of non-dominant B cell clones, or that some slight impairments in one of these mechanisms were present prior to malignancy detection, and may have even contributed to malignant transformation.

## 4 Discussion

CLL is a chronic disease, and M-CLL tumor clones may accumulate mutations in their IgHV genes for many years. For these reasons, we assumed that dominant clones would show different mutation characteristics than healthy control clones. Messmer and colleagues, who performed sequence-based mutational analysis of representative CLL IgHV gene sequences from the dominant clones of 172 CLL patients, found that dominant CLL sequences include more mutations than non-dominant ones (54); Petrova et al. used isotype-resolved BCR sequencing and indicated a distinct evolution of malignant CLL clones relative to clones from healthy volunteers (55). However, neither study characterized these mutations. Using IgHV lineage tree-based analyses, we found that dominant CLL clones undergo – or retain – more IgHV replacement mutations that alter amino acid physico-chemical properties than non-dominant or healthy control clones. Supporting the mutation analysis results, dominant CLL clone lineage trees possess tumor-typical, highly branched topologies, which correlate with weaker – but present – selection.

Since it is difficult to distinguish between the effects of impairments in SHM *vs.* selection, we used the focused binomial test, which to our knowledge is the only correct test for selection used on lymphomas to date, and found that CLL dominant clones undergo almost no selection *for* replacement mutations in their IgHV gene CDRs. However, dominant clones clearly maintain some selection *against* replacement mutations in their FWRs, although this selection is weaker than that observed in normal healthy controls. Similar alterations in IgHV selection were also found in our studies of other Ig gene mutating B cell malignancies (24, 29, 30). Our finding that CLL clones retain the selection against replacement mutations in their IgHV FWRs indicates a need for IgH transcription, translation, and proper protein folding, and agrees with previous studies showing that CLL tumor clones depend on some type of signals from the BCR complex (56–59).

Several IgHV repertoire studies used ML for classification – to discriminate between IgHV in tumors and those in normal tissues (60), to discriminate between IgHV from celiac patients and healthy individuals (61), or to classify relapsing-remitting multiple sclerosis IgHV CDR3 data from other neurological disease data (62). Here, we used an extensive list of lineage tree-based mutation characteristics to build ML models that could identify minor differences between non-dominant (presumed non-malignant) clones in CLL patients and healthy control trees. Ignoring the dominant clone data, we used ML to look for CLL patient-specific (rather than tumor-specific) impairments in SHM or antigen-driven selection; such information may yield targets for molecular research into what pre-disposes people for CLL and possible other lymphomas. The best ML model classified the non-dominant and healthy control trees with high accuracy, and indicated that CLL non-dominant clones have more transition mutations relative to healthy control clones. Messmer et al. indicated in 2004 that dominant clone CLL IgHV sequences show preference for transitions over transversions (54); our analysis shows for the first time that this preference exists even in the CLL *non*-dominant sequences. Although the non-dominant clones we included in this study were all small, we cannot exclude the possibility that some of the non-dominant clones we did include were also malignant. There could either be branches of the main tumor that have mutated so far away from it that our algorithm couldn’t identify them as related to the main clone, or unrelated CLL or different tumors in the same patient. However, at least some of the non-dominant clones must have been from normal B cells, so we believe that this finding is worth looking into. It is possible that the balance between transitions, which are created *via* simple replication over AID-introduced uracils, and transversions, which are created by several other DNA repair mechanisms, is disrupted in CLL patients (63–65), and that this disruption is somehow linked to the malignancy.

In summary, we present IgHV sequence lineage tree-based analysis of 15 M-CLL patient tumors, in comparison with the same patients’ non-dominant and with healthy control B cell clones, and show for the first time that (a) selection *against* replacement mutations is impaired in, but not completely abolished in the FWRs of, CLL dominant clones; SHM mechanisms may also be impaired in some way in CLL clones. (b) Even the non-dominant clones in CLL patients differ from those of healthy controls in various ways, the most notable being that they express higher fractions of transition mutations than healthy control clones. Performing a similar but larger scale study will allow a better understanding of IgHV SHM and selection in M-CLL, and may shed light on the clinical significance of the heterogeneity of M-CLL; the same methods would also be useful for studying any other tumor-related evolutionary processes that can be studied using lineage trees.

## Data availability statement

Raw sequence data used for analysis in this study are publicly available at the NCBI Sequencing Read Archive (www.ncbi.nlm.nih.gov/sra) under BioProject number PRJNA887723.

## Author contributions

HN designed the research, analyzed the data and wrote the manuscript. JA analyzed the healthy control data. OB and MK contributed the experimental data. RM and MK designed and supervised the research and wrote the manuscript. All authors contributed to the article and approved the submitted version.
