# Supplementary material for: B cell M-CLL clones retain selection against replacement mutations in their immunoglobulin gene framework regions
Source: Front Oncol. 2023 Mar 16;13:1115361. doi: 10.3389/fonc.2023.1115361 (PMC10060519; doi:10.3389/fonc.2023.1115361)

B cell CLL Dominant Clones Undergo Somatic Hypermutation and Selection Differently from Healthy Control B cell Clones

Hadas Neuman, Jessica Arrouasse, Ohad Benjamini, Ramit Mehr and Meirav Kedmi

Supplementary Figures

Figure S1. Lineage tree figures. (A) Sample dominant clone lineage trees. Due to their sizes, we had to choose the smallest ones, and even those figures had to be split into partly overlapping segments. (B) Sample non-dominant clone lineage trees of various sizes. (C) Sample healthy control clone lineage trees of various sizes, from the largest to the smallest. All notations and technical details are the same as in Figure 1.

A. Sample index#2:

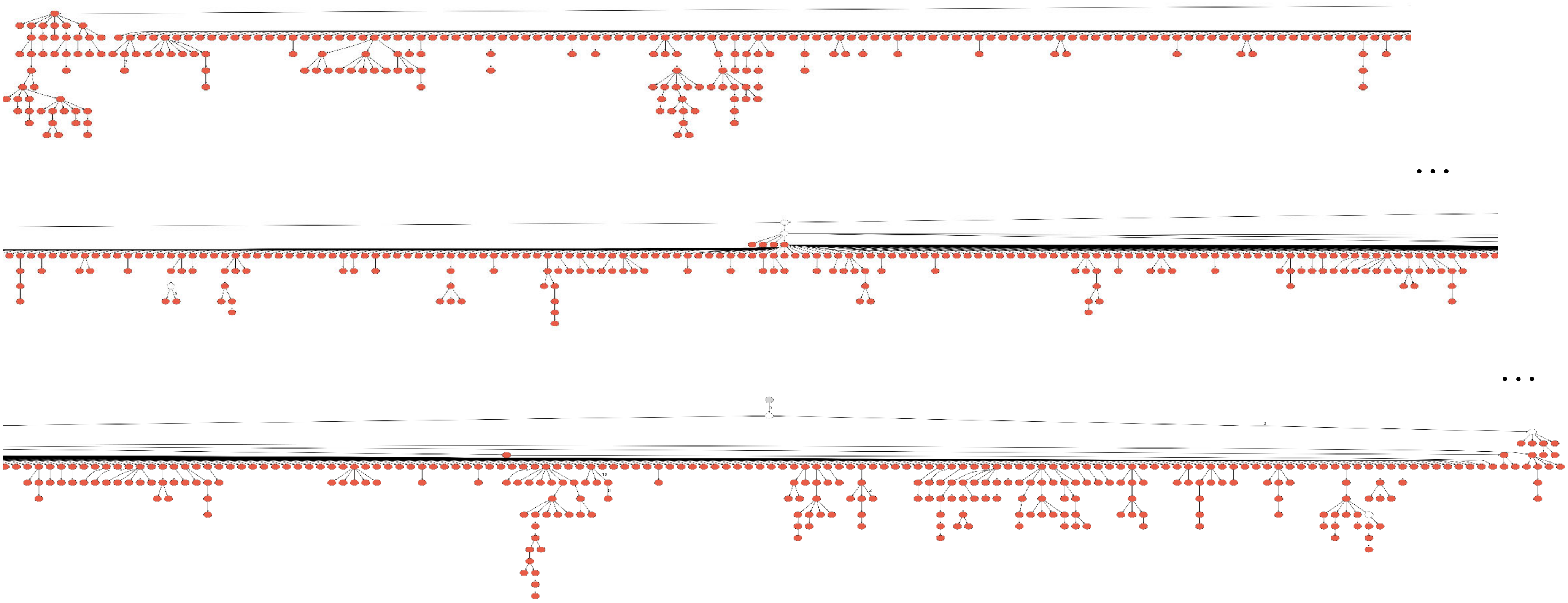

Sample index#3:

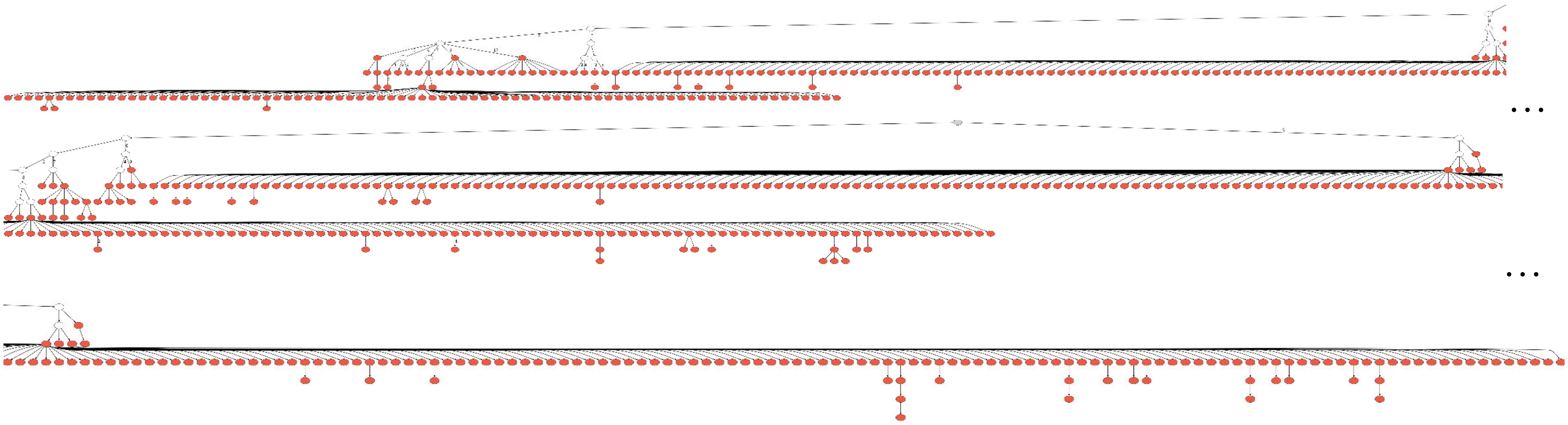

Sample index#6:

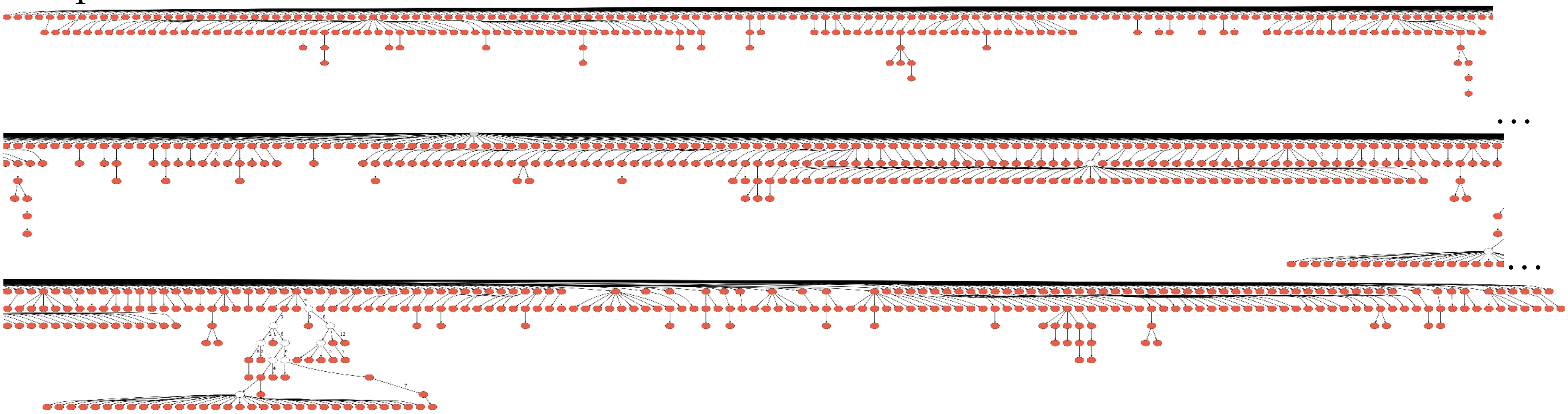

Sample index#14:

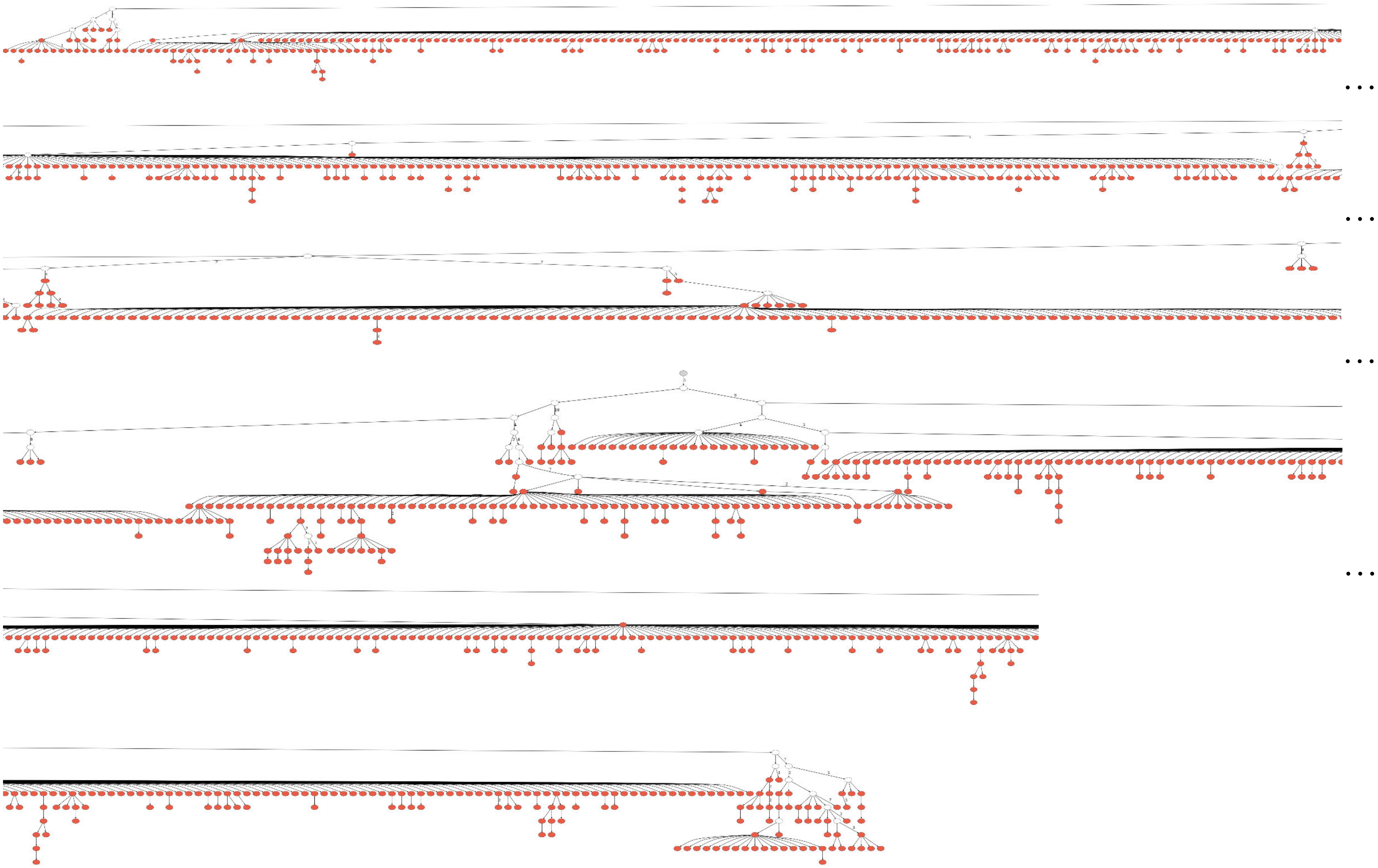

B.

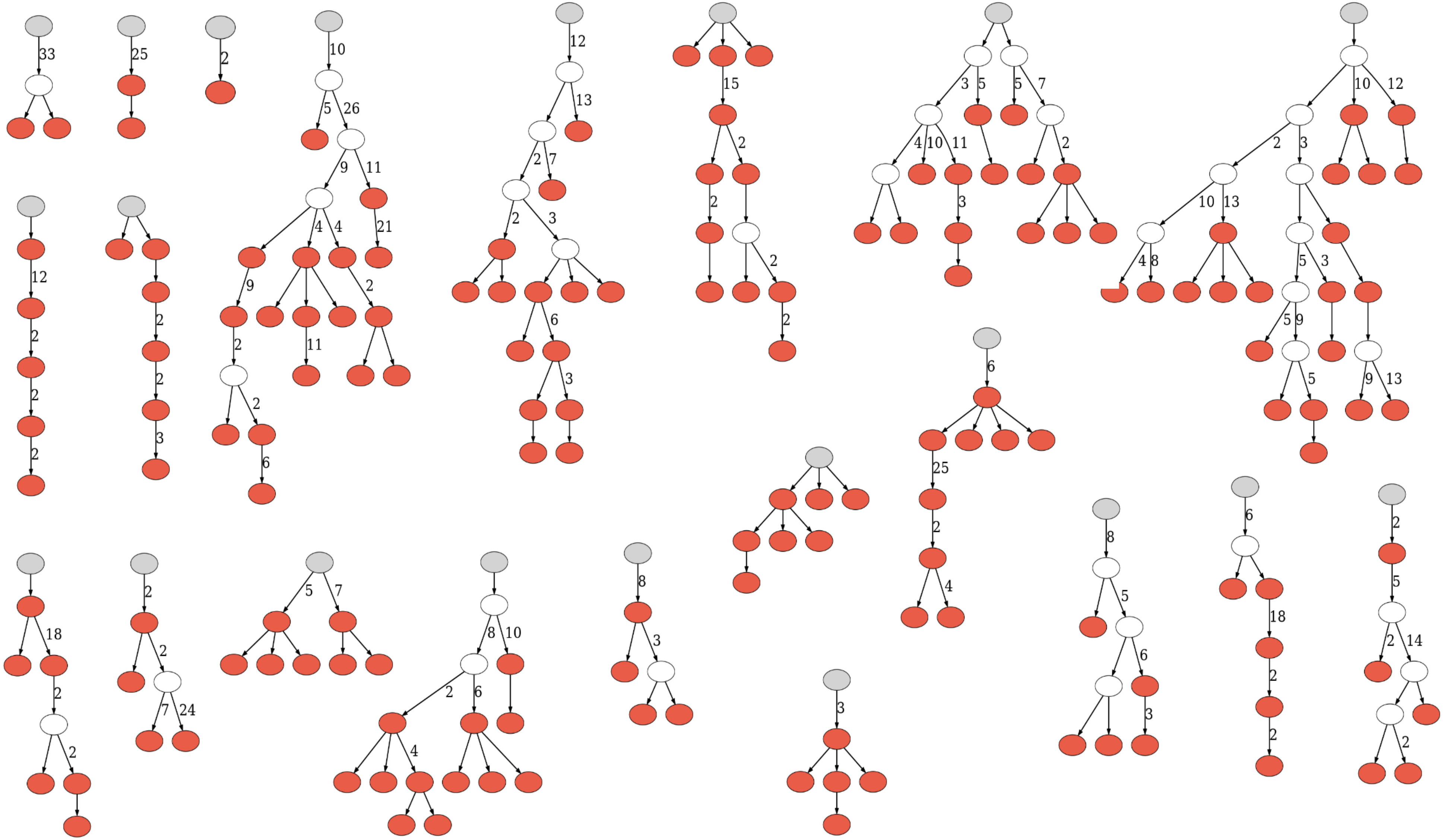

C.

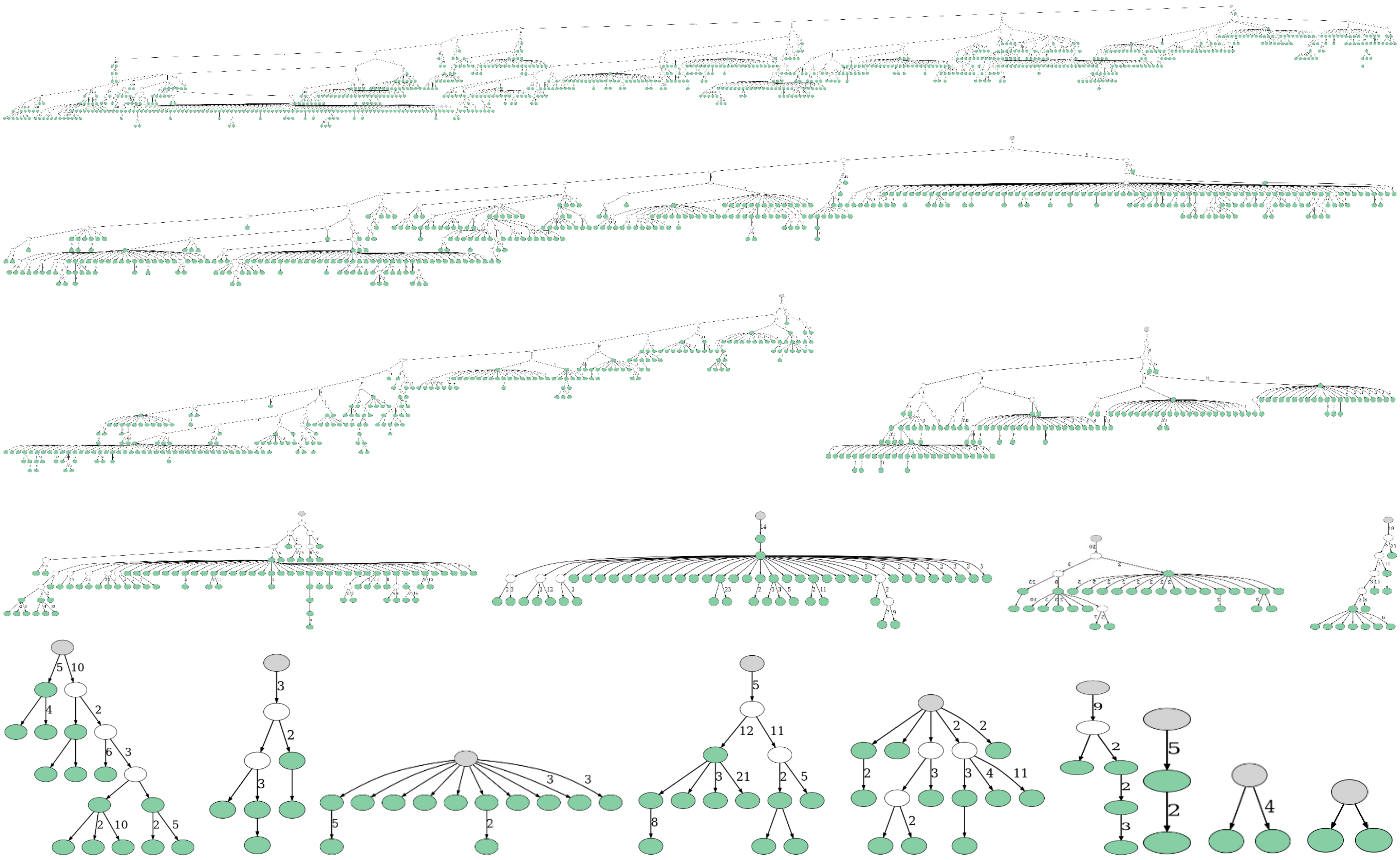

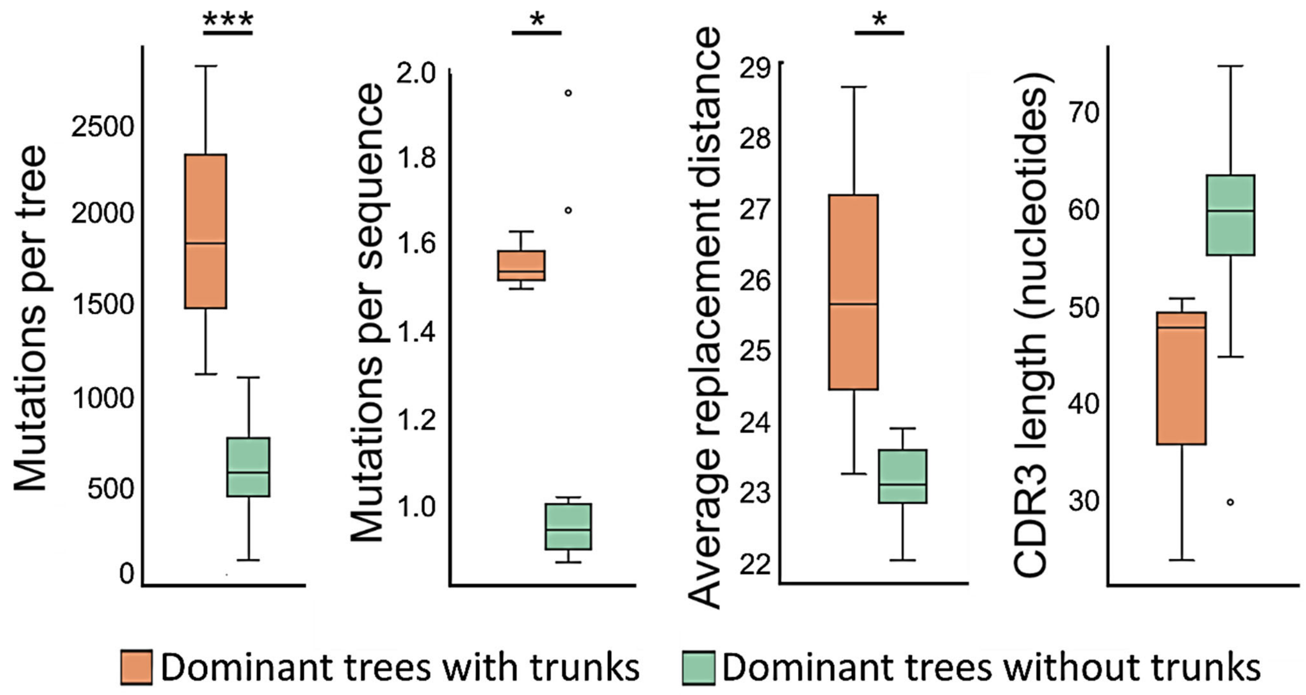

Figure S2. Originally trunkless dominant trees undergo somatic hypermutation and selection differently from dominant trunk-including trees. Compare with Figure 2. Student's T-test or Mann-Whitney test, $*p < 0.05$, $***p < 0.001$.

Figure S3. Dominant CLL lineage tree topologies suggest that CLL dominant clones retain some sensitivity to selection. (A) Trunk length, minimum root to leaf distance and minimum root to fork distance for trees with trunks. (B) Numbers of leaves, numbers of children emerging from the root and average numbers of children per node for trees with trunks. (C) Minimum root to leaf distance and minimum root to fork distance for trunkless trees. (D) Numbers of leaves, numbers of children emerging from the root and average numbers of children per node for trunkless trees. Only three dominant clone trees had trunks that could be removed for the trunkless analysis, and all three's roots had two children each, hence the lack of variability in this number (D, middle). The Paired T-test or the Wilcoxon paired test were used when comparing between dominant and non-dominant clones in the same patients, and the Student's T-test or the Mann-Whitney test – between patient and healthy control clones, depending on whether the data were distributed normally or not $*p < 0.05$, $**p < 0.01$, $***p < 0.001$.

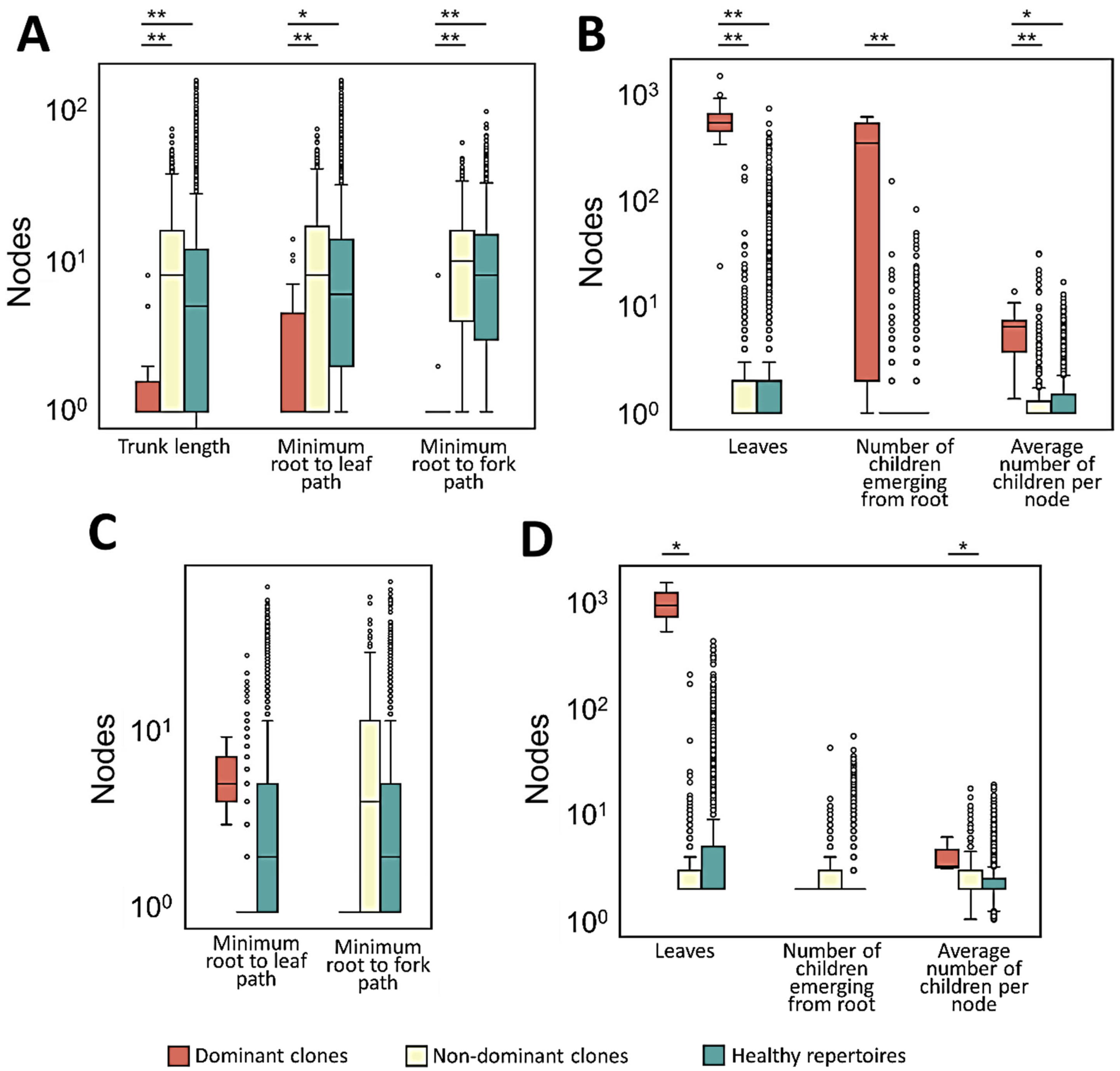

Supplement: Supplementary file 1 [file DataSheet_1.pdf]
